# Accelerated High Fidelity Prion Amplification Within and Across Prion Species Barriers

**DOI:** 10.1371/journal.ppat.1000139

**Published:** 2008-08-29

**Authors:** Kristi M. Green, Joaquín Castilla, Tanya S. Seward, Dana L. Napier, Jean E. Jewell, Claudio Soto, Glenn C. Telling

**Affiliations:** 1 Department of Microbiology, Immunology and Molecular Genetics, University of Kentucky, Lexington, Kentucky, United States of America; 2 Department of Neurology, University of Texas Medical Branch, Galveston, Texas, United States of America; 3 Sanders Brown Center on Aging, University of Kentucky, Lexington, Kentucky, United States of America; 4 Department of Veterinary Sciences, University of Wyoming, Laramie, Wyoming, United States of America; 5 Department of Neurology, University of Kentucky, Lexington, Kentucky, United States of America; University of Edinburgh, United Kingdom

## Abstract

Experimental obstacles have impeded our ability to study prion transmission within and, more particularly, between species. Here, we used cervid prion protein expressed in brain extracts of transgenic mice, referred to as Tg(CerPrP), as a substrate for in vitro generation of chronic wasting disease (CWD) prions by protein misfolding cyclic amplification (PMCA). Characterization of this infectivity in Tg(CerPrP) mice demonstrated that serial PMCA resulted in the high fidelity amplification of CWD prions with apparently unaltered properties. Using similar methods to amplify mouse RML prions and characterize the resulting novel cervid prions, we show that serial PMCA abrogated a transmission barrier that required several hundred days of adaptation and subsequent stabilization in Tg(CerPrP) mice. While both approaches produced cervid prions with characteristics distinct from CWD, the subtly different properties of the resulting individual prion isolates indicated that adaptation of mouse RML prions generated multiple strains following inter-species transmission. Our studies demonstrate that combined transgenic mouse and PMCA approaches not only expedite intra- and inter-species prion transmission, but also provide a facile means of generating and characterizing novel prion strains.

## Author Summary

Prions are unique pathogens that result from conversion of a normal host-encoded prion protein, PrP^C^, into a self-propagating, disease-associated conformation, referred to as PrP^Sc^. An important aspect of prion diseases is their transmissibility, frequently as epidemics. The contagious transmission of chronic wasting disease (CWD) of deer and elk is of particular concern. The elements governing prion transmission between species, including the influence of agent strain properties, remain enigmatic, in large part because of considerable difficulties associated with experimental manipulation of prions. The aim of this study was to evaluate the feasibility of expediting studies of intra- and inter-species prion transmission. We made use of transgenic mice as a source of deer prion protein for the production of CWD prions by protein misfolding cyclic amplification (PMCA). Characterization of infectivity in the same transgenic mice demonstrated that PMCA results in the efficient amplification of CWD prions with unaltered strain characteristics. Also, whereas adaptation of mouse prions to form novel cervid prions required several hundred days and subsequent stabilization in transgenic mice, we show that PMCA rapidly abrogated this inter-species transmission barrier. Our results indicate that PMCA can be used to replace the process of prion strain adaptation and selection occurring in vivo.

## Introduction

Prion diseases are transmissible, fatal, and incurable neurodegenerative disorders of the central nervous system (CNS) that include bovine spongiform encephalopathy (BSE), ovine scrapie, chronic wasting disease (CWD) of cervids and human Creutzfeldt-Jakob disease (CJD). While inoculation of diseased brain material into individuals of the same species typically reproduces disease, studies of prion transmissions are complicated by prolonged, clinically silent incubation periods lasting months to years. Inter-species prion transmission is generally an even less efficient process, a phenomenon referred to as the species barrier [1].

While studies in transgenic (Tg) mice [2]–[6] and cell-free systems [7], demonstrated the influence of PrP primary structure on prion transmission, agent strain properties are an equally important determinant. Thus, the time between inoculation and onset of clinical signs, referred to as the incubation time, is a parameter that varies between strains. Different strains may also induce distinct clinical signs in inoculated animals. Neuropathologically, strains are distinguished by reproducible differences in the distribution of spongiform degeneration of the cerebral grey matter, and by the deposition of PrP^Sc^, occasionally in the form of amyloid plaques. While the different strain properties of conventional pathogens are genomically encoded, it is less clear how multiple disease phenotypes can be accommodated in the context of a ‘protein only’ mechanism of pathogenesis where the infectious agent lacks nucleic acid. Numerous studies suggest that strain diversity is enciphered in the higher order structure of PrP^Sc^ [8]–[12]; accordingly, the biochemical properties of PrP^Sc^ have also been used as a means of typing prion isolates [13],[14].

While prion strain properties are stably maintained upon passage within a particular species, inter-species prion transmission may result in the acquisition of new strain properties, the most profound of which may be host range alteration [15],[16]. Thus, the species tropism of novel prion strains currently cannot be predicted. A powerful demonstration of the unpredictable influence of prion strains on species barriers is highlighted in the case of BSE. Cattle feed derived from rendered meat and bone meal which was contaminated with prions, possibly originating from scrapie-infected sheep, is the suspected origin of BSE [17]. BSE-related prion diseases were subsequently identified in domestic and captive wild cats [18],[19] and exotic ungulates. The recognition that a variant of CJD (vCJD) is caused by the BSE prion strain [20]–[23] raised major public health concerns. Like BSE, the origin of transmissible mink encephalopathy (TME) of ranch-raised mink, is thought to be prion-contaminated feed [24].

Recent years have witnessed the emergence of additional novel mammalian prion strains. Atypical scrapie is a recently-recognized and surprisingly prevalent prion disease of sheep of unknown origin and host-range. First reported in Norwegian sheep in 2003 and referred to as Nor98 [25], atypical scrapie appears to be a single, unique scrapie strain [26],[27] infecting sheep with *PRNP* genotypes usually associated with resistance to classical scrapie. The increasing geographic range, contagious transmission, uncertain strain prevalence, and environmental persistence of CWD are also of concern. Uncontrolled prion dissemination in wild cervid populations brings into question the risk of transmission to other species, for example via shared grazing of CWD-contaminated rangeland. Insights into the factors controlling prion transmission and host-range adaptation are clearly of paramount importance for containing further prion epidemics.

The aim of this study was to evaluate the feasibility of expediting studies of intra-, and inter-species prion transmission by combining the resources of protein misfolding cyclic amplification (PMCA) [28] with Tg mouse models of prion disease. During PMCA, the normal form of PrP, referred to as PrP^C^, is converted into protease-resistant PrP using small amounts of infectious PrP^Sc^. Continued recruitment and conversion of PrP^C^ by PrP^Sc^ is accomplished by sonication in a process analogous to amplification of DNA by the polymerase chain reaction [29]. We previously showed that Tg mice expressing PrP from mule deer, referred to as Tg(CerPrP) mice, are susceptible to prions from deer and elk dying of CWD [30]–[32]. Several other groups subsequently confirmed these observations using similar mouse models [33]–[37]. Here we used cervid PrP^C^ (CerPrP^C^) expressed in the brains of Tg mice for the generation of CWD prions by PMCA. Using Tg(CerPrP) mice to characterize this in vitro-generated infectivity we demonstrate that PMCA results in the high fidelity amplification of CWD prions with apparently unaltered strain properties. In addition, while adaptation of mouse prions to form novel cervid prions required several hundred days in Tg(CerPrP) mice, we show that PMCA abrogated this barrier to prion transmission resulting in the rapid generation of novel cervid prions with similar properties.

## Results

### Serial PMCA of CWD-seeded CerPrP^C^ from Tg(CerPrP) mice results in high fidelity CWD prion replication

**Figure 1:**
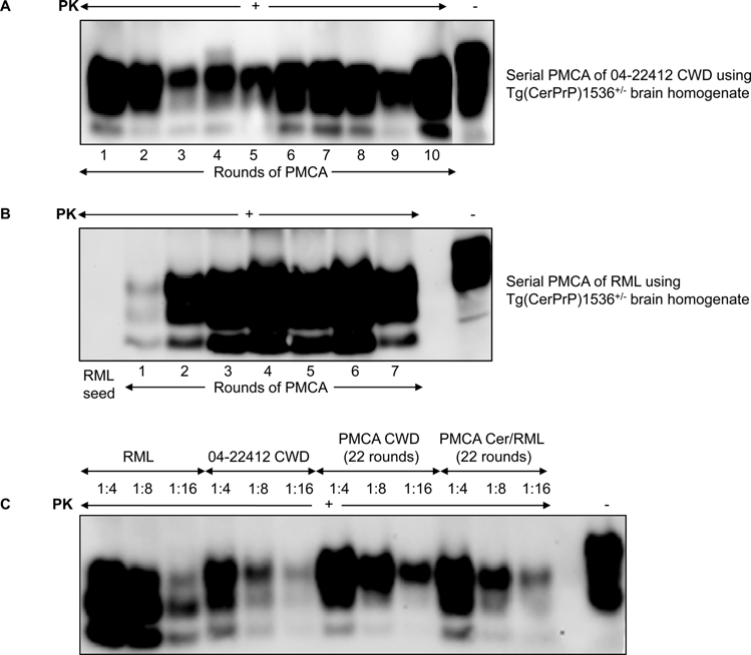
Western blot analysis showing amplification of protease-resistant CerPrP by serial PMCA. A: Serial PMCA of 04-22412 CWD using Tg(CerPrP)1536^+/−^ brain homogenate. CWD prions in a 10% brain homogenate of diseased mule deer 04-22412 were diluted 10-fold into 10% brain homogenate from perfused Tg(CerPrP)1536^+/−^ mice. Following a round of PMCA, the sample, containing amplified protease-resistant CerPrP, was diluted 10-fold into 10% brain homogenate from perfused Tg(CerPrP)1536^+/−^ mice for a further round of PMCA. This process of serial PMCA was repeated for 22 rounds. PK-treated samples from each of the first 10 rounds were analyzed by Western blotting. In the final lane, a sample from Tg brain homogenate without PK treatment was loaded. B: Serial PMCA of RML using Tg(CerPrP)1536^+/−^ brain homogenate. Mouse RML prions in a 10% brain homogenate from a diseased wild type FVB mouse were diluted 10-fold into 10% brain homogenate from perfused Tg(CerPrP)1536^+/−^ mice. Serial PMCA was repeated for 22 rounds. PK-treated samples from each of the first 7 rounds were analyzed by Western blotting. The unamplified RML seed that produced protease-resistant PrP following PMCA in round 1 was loaded in the first lane, while a sample from Tg brain homogenate without PK treatment was loaded in the final lane. C: Western blot quantification of protease-resistant PrP in inocula used to challenge Tg(CerPrP)1536^+/−^ mice. Samples were PK-treated as indicated. Ratios indicate the fold dilution of the original preparation. In the final lane, a sample from CWD brain homogenate without PK treatment was loaded.

A PMCA reaction was established using a CWD prion seed in a 10% brain homogenate from diseased mule deer 04-22412, diluted 10-fold into 10% brain homogenate prepared from perfused Tg(CerPrP)1536^+/−^ mice [30]. Following a round of PMCA consisting of alternating periods of sonication and incubation for 36 cycles, the product, which contained amplified protease-resistant CerPrP (Fig. 1A), was diluted 10-fold into another reaction containing CerPrP^C^ from Tg(CerPrP)1536^+/−^ mouse brain homogenate for a further round of PMCA. This process of serial PMCA was repeated for 22 rounds. In accordance with previous studies using the experimentally-adapted hamster scrapie isolate 263K [38],[39], and the experimentally-adapted Chandler scrapie isolate [40], protease-resistant CerPrP was amplified to high levels during each round of serial PMCA (Fig. 1A and C). In contrast, after 10 rounds of serial PMCA of six duplicated samples of a healthy Tg(CerPrP)1536^+/−^ brain extract, no protease-resistant PrP was produced in the absence of prion seeds (data not shown).

To ascertain whether this process resulted in the in vitro amplification of CWD prions, Tg(CerPrP)1536^+/−^ mice were intracerebrally challenged with the product of 22 rounds of serial PMCA (Fig. 1C). A separate cohort was inoculated with a 1% brain homogenate of the CWD-infected 04-22412 mule deer isolate that was the seed for the initial round of PMCA. In both cases, Tg(CerPrP)1536^+/−^ mice were inoculated with preparations containing similar amounts of protease-resistant CerPrP, as determined by Western blot analysis (Fig. 1C). Serial PMCA reactions initially seeded with 04-22412 CWD prions but using *Prnp^0/0^* knockout instead of Tg(CerPrP)1536^+/−^ mouse brain homogenate were also performed in parallel. A cohort of Tg(CerPrP)1536^+/−^ mice inoculated with this material after 22 rounds of serial PMCA served as negative controls to show that the original CWD inoculum was not detectable.

**Figure 2:**
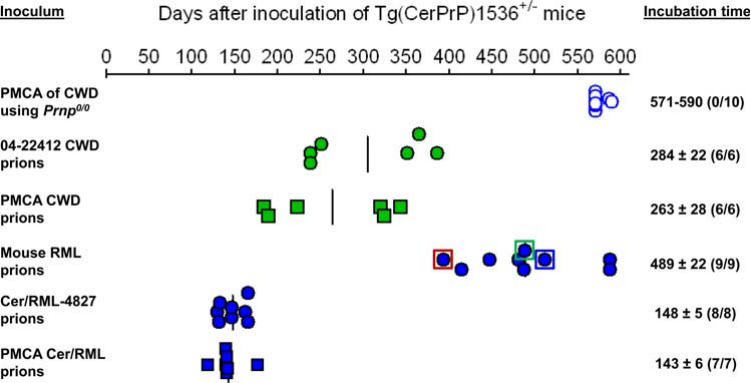
Generation and characterization of PMCA-derived prions using Tg(CerPrP)1536^+/−^ mice. Each symbol represents an individual mouse. Closed symbols indicate diseased mice and open symbols indicate asymptomatic mice. Green symbols indicate prions originating from CWD; blue symbols indicate prions originating from RML; circles indicate in vivo-derived prions; squares indicate PMCA-derived prions. The blue circle surrounded by the red square signifies mouse #4827 that was the origin of Cer/RML-4827 prions, while the green and blue squares signify mice #5302 and #4825 respectively, the brains of which were analyzed by histoblotting. Incubation times are expressed as the mean±standard error of the mean; listed in parenthesis is number of diseased mice/ number of mice inoculated.

**Figure 3:**
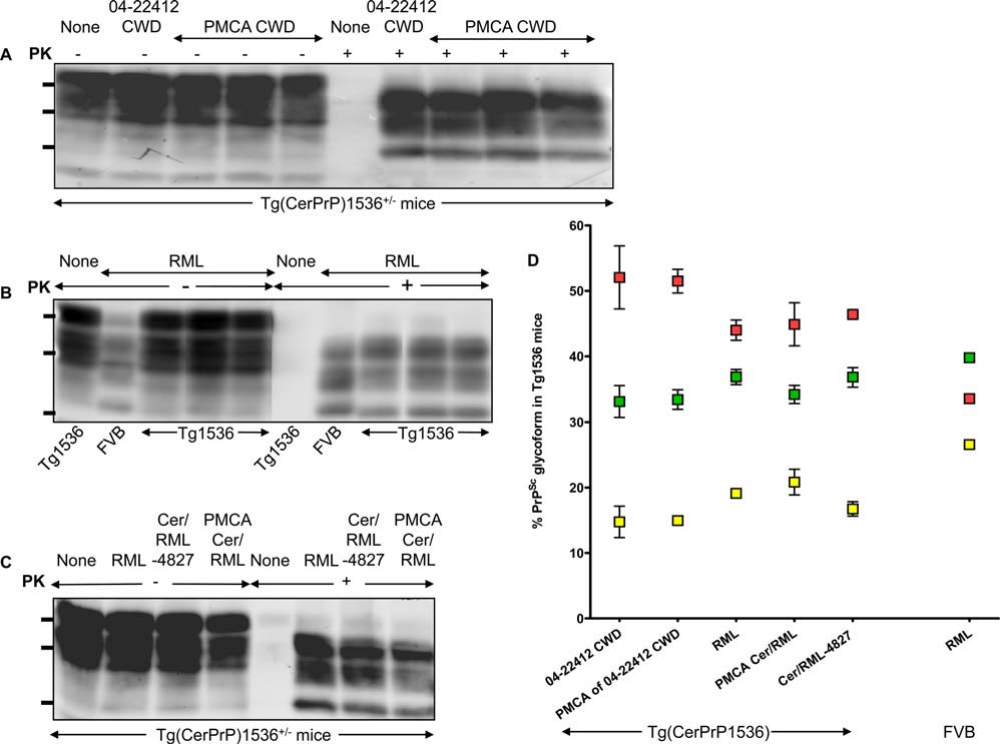
Characteristics of PrP^Sc^ produced in Tg(CerPrP)1536^+/−^ mice. A: Western blot showing accumulation of CerPrP^Sc^ in the brains of diseased Tg(CerPrP)1536^+/−^ mice inoculated with CWD or PMCA CWD prions. B: Western blot comparison of PrP^Sc^ in the brains of diseased FVB and Tg(CerPrP)1536^+/−^ mice (Tg1536) inoculated with mouse RML prions. C. Western blot showing CerPrP^Sc^ accumulation in Tg(CerPrP)1536^+/−^ mice infected with mouse RML prions, Cer/RML-4827, and PMCA Cer/RML prions. D: Ratio of three protease-resistant PrP glycoforms produced in the brains of diseased Tg(CerPrP)1536^+/−^ mice or FVB mice. Data points represent the mean relative proportions of di-, mono-, and un-glycosylated PrP as a percentage derived from densitometric quantification of PrP^Sc^ in brains of three individual diseased mice in each case. Error bars indicate the standard error of the mean which, in some cases, was smaller than the symbols used. Samples for Western blot analysis were either untreated (−) or treated (+) with PK and 50 µg and 100 µg of total protein was loaded for untreated and treated samples respectively. The positions of protein molecular mass markers at 37, 25 and 20 kDa (from top to bottom) are shown.

**Figure 4:**
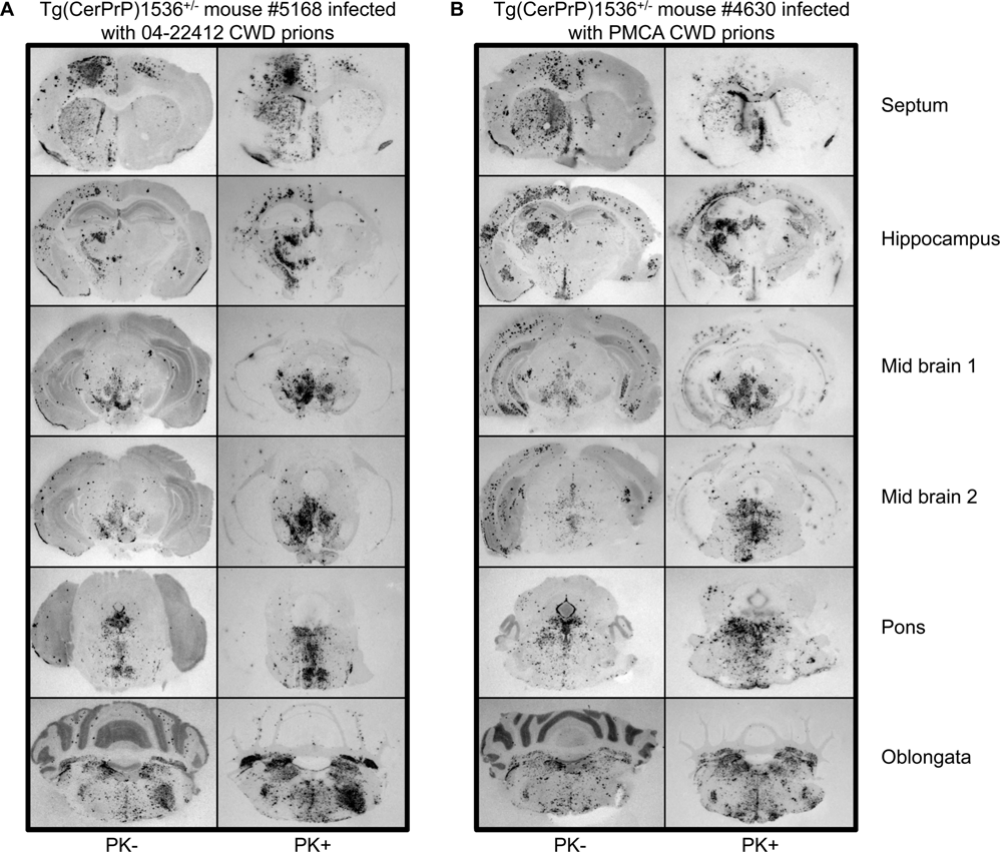
Regional distribution of CerPrP in the CNS of diseased Tg(CerPrP)1536^+/−^ mice infected with CWD or PMCA CWD prions. PK-treated (+) or untreated (−) histoblotted coronal sections, as indicated, of terminally sick Tg(CerPrP)1536^+/−^ mice inoculated with A, naturally occurring CWD prions from mule deer isolate 04-22412 or B, PMCA-derived CWD prions. Histoblots were stained with Hum-P anti-PrP recombinant Fab followed by alkaline phosphatase-conjugated goat anti-human secondary antibody.

**Figure 5:**
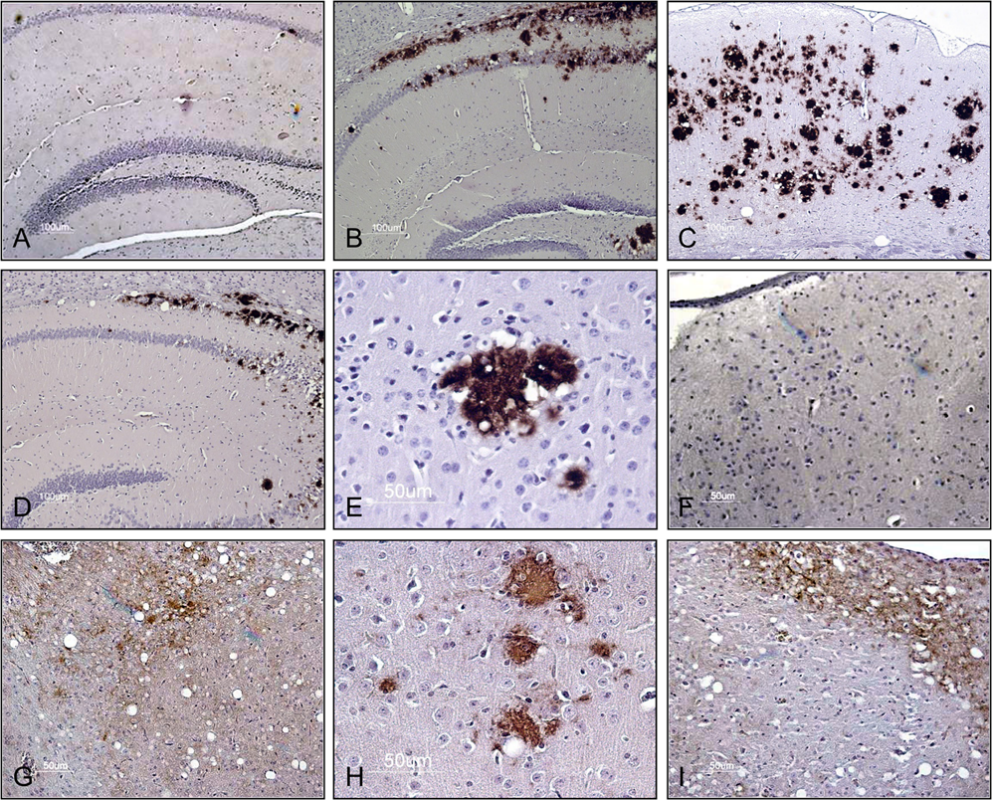
Immunohistochemical detection of CerPrP^Sc^ and spongiform degeneration in the brains of diseased Tg(CerPrP)1536^+/−^ mice. A, B, and D are sections through the hippocampus of non-diseased or diseased Tg(CerPrP)1536^+/−^ mice; section C is from the cerebral cortex. A, absence of spongiform pathology and immunohistochemically-reactive PrP in the hippocampus of an asymptomatic PBS-inoculated Tg(CerPrP)1536^+/−^ mouse; B, accumulation of plaques in the hippocampus of diseased Tg(CerPrP)1536^+/−^ mouse inoculated with naturally occurring 04-22412 CWD prions; C, accumulation of plaques in the cerebral cortex of a diseased Tg(CerPrP)1536^+/−^ mouse inoculated with naturally occurring 04-22412 CWD prions; D, accumulation of plaques in the hippocampus of diseased Tg(CerPrP)1536^+/−^ mouse inoculated with PMCA CWD prions; E, high magnification of a large plaque aggregate rimmed by vacuoles; F, absence of spongiform pathology and immunohistochemically reactive PrP in the medulla of asymptomatic PBS-inoculated Tg(CerPrP) 1536^+/−^ mouse; G, diffuse PrP accumulation in the medulla of diseased Tg(CerPrP)1536^+/−^ mouse #5297 inoculated with mouse RML prions; H, high magnification of a section thought the hippocampus of diseased Tg(CerPrP)1536^+/−^ mouse #5300 showing PrP accumulation in small plaques; I, diffuse PrP accumulation in the medulla of a diseased Tg(CerPrP)1536^+/−^ mouse inoculated with PMCA Cer/RML prions. Hematoxylin was used as counterstain. Bar = 100 µm in A–D; Bar = 50 µm in E–I.

All Tg(CerPrP)1536^+/−^ mice (n = 6) inoculated with material derived from serial PMCA of 04-22412 CWD prions using CerPrP^C^ from the brains of Tg(CerPrP)1536^+/−^ mice developed disease with a mean incubation time of 263±28 (mean±standard error) days (d) (Fig. 2). Consistent with previous results [30]–32, CWD prions from the brain of diseased 04-22412 mule deer also induced disease in Tg(CerPrP)1536^+/−^ mice (n = 6) with an incubation time of 284±22 d. The clinical signs that accompanied prion disease were identical in both cases, and included truncal ataxia and slowed movement, increased tone of the tail, dorsal kyphosis, head bobbing or tilting, and roughened coat. Confirming that prion infectivity produced by 22 rounds of serial PMCA was unrelated to persistence of the initial 04-22412 CWD prion seed, no disease was registered in Tg(CerPrP)1536^+/−^ mice inoculated with preparations from the negative control reaction in which 04-22412 CWD prions were seeded into *Prnp^0/0^* brain homogenate followed by 22 rounds serial PMCA (Fig. 2). Detection of CerPrP^Sc^ in the brains of diseased Tg(CerPrP)1536^+/−^ mice by Western blotting (Fig. 3A), histoblotting (Fig. 4), and immunohistochemical analysis (Fig. 5B–E) confirmed that the clinical signs following inoculation with CWD prions or the amplified samples were the consequence of prion disease. Collectively these results demonstrate that serial PMCA resulted in the efficient in vitro production of infectious CWD prions. We therefore refer to infectivity in the amplified samples as PMCA CWD prions.

### Concordant strain properties of PMCA-derived and naturally occurring CWD prions

In order to fully evaluate the biochemical and neuropathological characteristics of disease induced by both inocula, we performed detailed comparative studies of the brains of mice infected with naturally occurring and PMCA CWD prions. The similar incubation times of naturally occurring and PMCA CWD prions (Fig. 2) raised the possibility that the strain properties of the 04-22412 CWD prion isolate were maintained during serial PMCA. Consistent with this notion, the electrophoretic mobilities (Fig. 3A) and glycosylation profiles of CerPrP^Sc^ produced in the brains of Tg(CerPrP)1536^+/−^ mice inoculated with both preparations were similar (Fig. 3D).

Assessment of the neuroanatomical distribution of PrP^Sc^ by histoblotting is another parameter that has been used to characterize prion strains [20],[21],^32^,^41^,^42^. The appearance and distribution of CerPrP^Sc^ throughout histoblotted brain sections of diseased Tg(CerPrP)1536^+/−^ mice infected with 04-22412 CWD (Fig. 4A) or PMCA CWD prions (Fig. 4B) were similar (n = 1 in each group). Markedly punctate accumulations of CerPrP^Sc^ were present in histoblotted brain sections of mice infected with both naturally occurring and PMCA CWD prions, either prior to, or following treatment with proteinase K (PK). CerPrP^Sc^-containing aggregates often coalesced into larger immunoreactive structures. Similar aggregation and distribution of CerPrP^Sc^ has been reported in Tg(CerPrP)1536^+/−^ mice infected with various naturally occurring deer and elk CWD isolates [30],[32].

The accumulation of CerPrP^Sc^ in plaques was confirmed by immunohistochemical analyses of brains from diseased Tg(CerPrP)1536^+/−^ mice infected with naturally occurring CWD prions (Fig. 5B and C) and PMCA CWD prions (Fig. 5D and E). The distribution of immunoreactive plaques and accompanying spongiform degeneration was similar in both cases (Fig. 5B–E), with plaques often coalescing into larger structures frequently bordered by vacuoles (Fig. 5E).

**Figure 6:**
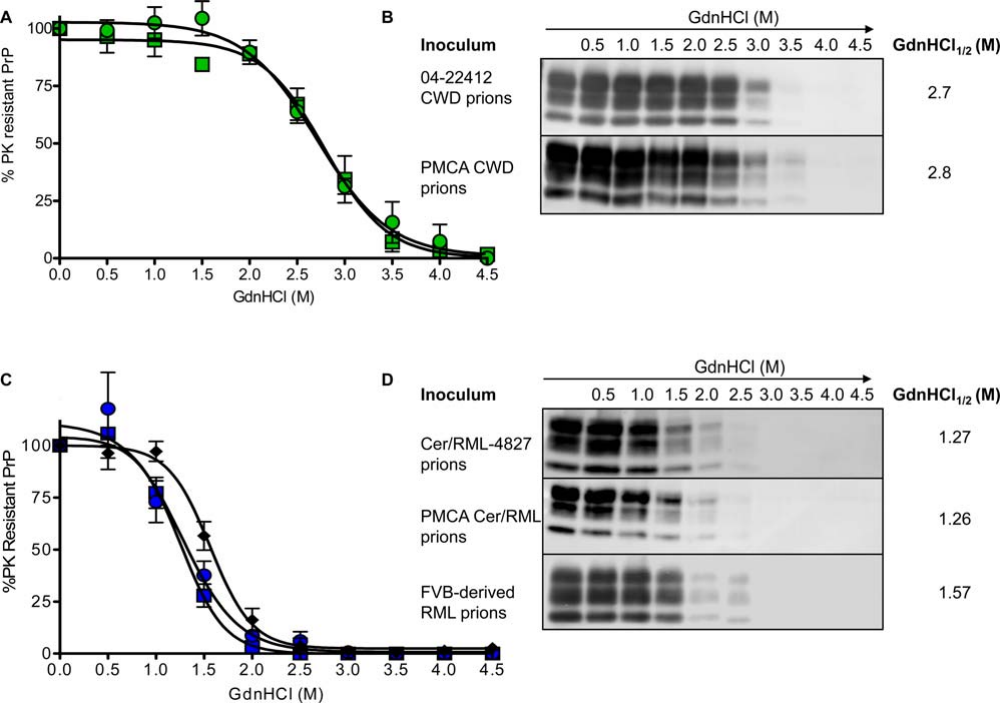
Assessment of the conformational stability of PrP^Sc^ in the brains of diseased mice. In A and C, densitometric analysis of immunoblots shows the percentage of protease-resistant CerPrP^Sc^ as a function of GdnHCl concentration. The sigmoidal dose-response was plotted using a four-parameter algorithm and non-linear least-square fit. Each point shown is the mean value derived from densitometric quantification of PK-resistant PrP in three diseased Tg(CerPrP)1536^+/−^ mouse brain extracts in each study group. Error bars indicate the standard error of the mean which, in some cases, was smaller than the size of the symbols used to indicate the mean. A, Tg(CerPrP)1536^+/−^ mice inoculated with 04-22412 CWD prions (green filled circles), or PMCA-derived CWD prions (green filled squares); C, Tg(CerPrP)1536^+/−^ mice inoculated with Cer/RML-4827 prions (blue filled circles), or PMCA Cer/RML prions (blue filled squares). For comparison, the conformational stability of MoPrP^Sc^ in the brains of wild type FVB mice infected with RML prions is shown (black diamonds). In B and D, representative immunoblots of protease-resistant PrP following PK treatment are shown. The mean GdnHCl_1/2_ value, representing the concentration at which half the PrP^Sc^ in each series was denatured, is also shown.

Previous studies showed the unfolding characteristics of PrP^Sc^ to be a sensitive and quantitative means of assessing strain-dependent differences in PrP^Sc^ conformation [11],[12],[32],[43]. We therefore determined the relative stabilities of CerPrP^Sc^ in the brains of Tg(CerPrP)1536^+/−^ mice infected with PMCA-generated or in vivo-derived CWD prions. Brian extracts were treated with increasing concentrations of guanidine hydrochloride (GdnHCl), followed by PK digestion and analysis of residual CerPrP^Sc^ by Western blotting. When plotted, the mean amounts of PK-resistant PrP in the brains of three diseased Tg(CerPrP)1536^+/−^ mice at each concentration of denaturant, formed sigmoidal curves. The transition point at the concentration where half the CerPrP^Sc^ in the samples was denatured is referred to as the mean GdnHCl_1/2_ value. Similar denaturation properties and mean GdnHCl_1/2_ values indicated comparable CerPrP^Sc^ stabilities following infection with 04-22412 CWD and PMCA CWD prions (Fig. 6A and B).

Collectively, the concordant clinical and histological profiles of Tg(CerPrP)1536^+/−^ mice infected with naturally occurring and PMCA-derived CWD prions, as well as the similar biochemical properties of the resulting CerPrP^Sc^, indicate that the characteristics of 04-22412 CWD prions were maintained during serial PMCA.

### Interspecies transmission of mouse RML prions in Tg(CerPrP) mice results in variable CNS disease

While deer and elk CWD prions propagated efficiently in Tg(CerPrP)1536^+/−^ mice, with 100% rates of transmission and mean incubation times ranging from ∼225 to 270 d, our previous studies showed that Tg(CerPrP)1536^+/−^ mice remained free of prion disease for >1 year after infection with mouse RML prions [30]. To fully characterize the extent of this transmission barrier, we challenged additional Tg(CerPrP)1536^+/−^ mice with mouse RML prions and extended our observations beyond one year. While all RML-inoculated Tg(CerPrP)1536^+/−^ mice (n = 9) eventually developed clinical signs, the time to disease onset was protracted and highly variable (mean incubation time, 489±22 d; range of disease onset ∼400 to 590 d) (Fig. 2). In contrast to the predominantly monoglycosylated profile of mouse PrP^Sc^ in the brains of RML infected wild type FVB mice (Figs. 1C, 3B and 3D), CerPrP^Sc^ produced in the brains of RML infected Tg(CerPrP)1536^+/−^ mice was predominantly diglycosylated (Fig. 3B, C and D). This suggested that adaptation of mouse RML prions occurred following transit across a species barrier in mice expressing CerPrP^C^.

**Figure 7:**
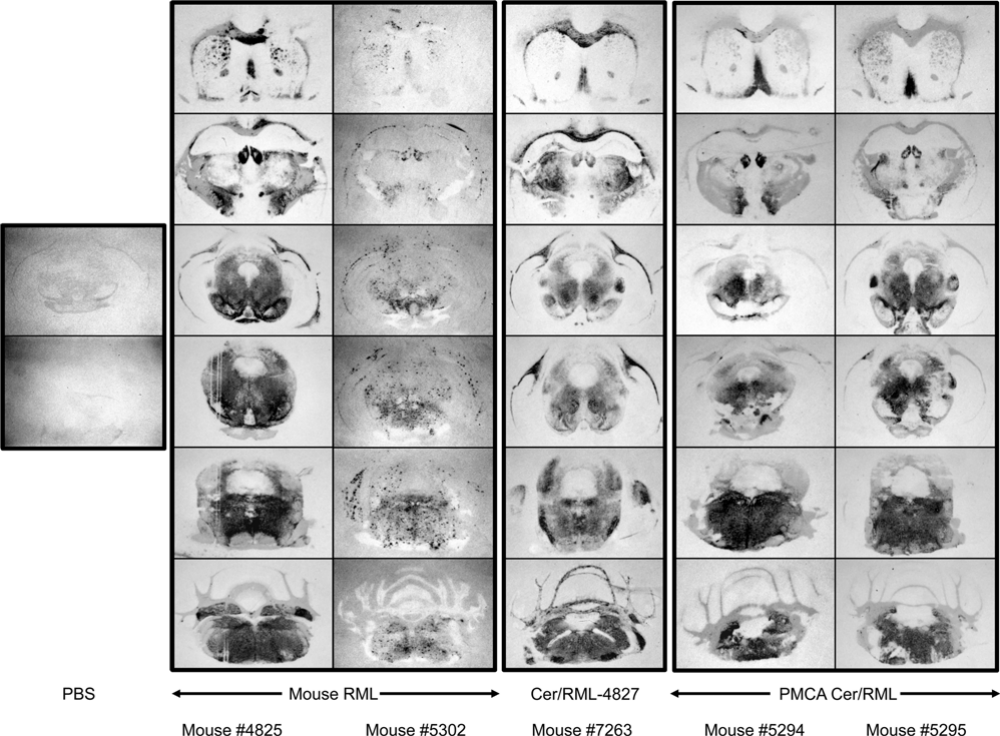
Regional distribution of CerPrP in the CNS of diseased Tg(CerPrP)1536^+/−^ mice infected with mouse RML prions, in vivo-adapted Cer/RML prions, and PMCA-adapted Cer/RML prions. PK-treated histoblotted coronal sections though, from top to bottom, the region of the septum, hippocampus, anterior midbrain, posterior midbrain, pons, and oblongata from diseased Tg(CerPrP)1536^+/−^ mice inoculated with mouse RML prions, Cer/RML-4827, or PMCA Cer/RML prions. Sections were prepared from two different Tg(CerPrP)1536^+/−^ mice inoculated with mouse RML prions (#4825 and #5302) and two different Tg(CerPrP)1536^+/−^ mice inoculated with PMCA Cer/RML prions (#5294 and #5295). Lateral areas of the posterior midbrain section of mouse #4825 were lost during tissue processing. Histoblots of sections through the anterior and posterior midbrain from an asymptomatic age-matched Tg(CerPrP)1536^+/−^ mouse inoculated with PBS, shown to the left, demonstrate the specificity of immunostaining with Hum-P anti-PrP recombinant Fab.

Histoblot analysis revealed variable distribution and aggregation of CerPrP^Sc^ in the CNS of two diseased Tg(CerPrP)1536^+/−^ mice infected with mouse RML prions (Fig. 7). The deposition of CerPrP^Sc^ in the brain of Tg(CerPrP)1536^+/−^ mouse #4825, which developed disease 512 d after infection, was widespread and diffuse. In contrast, CerPrP^Sc^ in the brain of Tg(CerPrP)1536^+/−^ mouse #5302, which developed disease after 488 d, accumulated in small, discrete plaques. The punctate staining observed in mouse #5302 differed from the CerPrP^Sc^ aggregates in Tg(CerPrP)1536^+/−^ mice infected with naturally occurring or PMCA CWD prions (Fig. 4), which were deposited in different brain regions, and frequently coalesced into larger immunoreactive structures. Immunohistochemical analysis of additional RML-infected Tg(CerPrP)1536^+/−^ mouse brains confirmed that CerPrP^Sc^ deposition and aggregation varied between animals. While diffuse CerPrP^Sc^ staining characterized the CNS of animal #5297 (Fig. 5G), CerPrP^Sc^ accumulated in small plaques in the CNS of animal #5300 (Fig. 5H).

### Abrogation of the transmission barrier to mouse prions in Tg(CerPrP) mice produces novel cervid-adapted prions with properties distinct from CWD

The brain of Tg(CerPrP)1536^+/−^ mouse #4827 that developed disease 394 d after infection with mouse RML prions was prepared for serial transmission studies. Infectivity in the brain of this mouse, referred to as Cer/RML-4827 prions, induced disease in Tg(CerPrP)1536^+/−^ mice (n = 8) with a mean incubation time of 148±5 d (Fig. 2). This substantial reduction in time to onset of disease, as well as the narrow range of incubation times on second passage, is characteristic of prion adaptation following transit across a species barrier. The diglycosylated CerPrP^Sc^ pattern that characterized infection of Tg(CerPrP)1536^+/−^ mice with mouse RML prions was maintained upon passage of Cer/RML-4827 prions to Tg(CerPrP)1536^+/−^ mice (Fig. 3C and D). Histoblot analysis of a recipient Tg(CerPrP)1536^+/−^ mouse brain #7263 showed that infection with Cer/RML-4827 prions was characterized by diffuse rather than punctate CerPrP^Sc^ distribution (Fig. 7).

The denaturation profile of CerPrP^Sc^ in the brains of Tg(CerPrP)1536^+/−^ mice infected with Cer/RML-4827 prions differed from mouse PrP^Sc^ in the brains of RML-infected FVB mice (mean GdnHCl_1/2_ values of 1.27 M and 1.57 M respectively, Fig. 6C and D). This conformational difference is consistent with adaptation of mouse RML prions following replication in Tg(CerPrP)1536^+/−^ mice. Moreover, the denaturation profile of CerPrP^Sc^ produced in response to infection with Cer/RML-4827 prions was considerably different from the profiles of CerPrP^Sc^ produced following infection with naturally occurring or PMCA-derived CWD prions (mean GdnHCl_1/2_ values of 1.27 M and 2.7–2.8 M respectively, Fig. 6A and B). This indicated that adaptation of mouse RML prions in Tg(CerPrP)1536^+/−^ mice resulted in the formation of novel cervid prions with a conformation distinct from CWD.

### Formation and characterization of PMCA-derived cervid adapted RML prions

We investigated whether PMCA could abrogate the barrier to mouse RML prion transmission that was ultimately breached following adaptation in Tg(CerPrP)1536^+/−^ mice. Brain homogenates from uninfected Tg(CerPrP)1536^+/−^ mice were seeded with mouse RML prions and 22 rounds of serial PMCA were performed as before. Protease-resistant CerPrP was amplified to high levels during each round of serial PMCA, culminating in 22 rounds (Fig. 1B and C). The change in PrP^Sc^ glycoform pattern that occurred following passage of mouse RML prions from wild type to Tg(CerPrP)1536^+/−^ mice (Fig. 3B and D) also appeared to be a feature of RML adaptation during serial PMCA, with protease-resistant CerPrP becoming predominantly diglycosylated at round 2 and thereafter (Fig. 1B). No protease-resistant PrP was produced in the absence of prion seeds after 10 rounds of serial PMCA of six duplicated samples of a healthy Tg(CerPrP)1536^+/−^ brain extract (data not shown).

Remarkably, inoculation of Tg(CerPrP)1536^+/−^ mice with this PMCA-adapted material, rapidly induced disease in all inoculated Tg(CerPrP)1536^+/−^ mice (n = 7). The 143±6 d mean incubation time was strikingly similar to the ∼150 d mean incubation time of Cer/RML-4827 prions that resulted from adaptation of RML prions following replication in Tg(CerPrP)1536^+/−^ mice (Fig. 2). This indicated that the barrier to inter-species transmission of mouse RML prions, which requires several hundred days of adaptation in Tg(CerPrP) mice and stabilization on serial passage, can be directly bypassed by serial PMCA of RML using CerPrP^C^ from Tg mouse brain. We therefore refer to infectivity in this amplified sample as PMCA Cer/RML prions.

Using Western blotting, conformational stability assays, and histoblotting we characterized the properties of CerPrP^Sc^ produced in the brains of diseased Tg(CerPrP)1536^+/−^ mice infected with PMCA Cer/RML prions. The diglycosylated profile of CerPrP^Sc^ in the PMCA Cer/RML inoculum (Fig. 1C) was maintained in the brains of diseased Tg(CerPrP)1536^+/−^ mice (Fig. 3C and D). The denaturation profile and mean GdnHCl_1/2_ value of CerPrP^Sc^ in the brains of Tg(CerPrP)1536^+/−^ mice infected with PMCA Cer/RML prions was equivalent to Cer/RML-4827, but different from RML in wild type FVB mice or from naturally occurring or PMCA-derived CWD prions in Tg(CerPrP)1536^+/−^ mice. This indicated that, similar to the adaptation of RML in Tg(CerPrP)1536^+/−^ mice, serial PMCA resulted in adaptation of mouse RML prions to produce novel cervid prions with a CerPrP^Sc^ conformation distinct CWD prions. Histoblotting revealed a consistent pattern of CerPrP^Sc^ distribution in the brains of two Tg(CerPrP)1536^+/−^ mice infected with PMCA Cer/RML prions (Fig. 7). The diffuse CerPrP^Sc^ deposition in the brains of two such mice, referred to as #5294 and #5295, was distinct from the small punctate staining pattern in the CNS of Tg(CerPrP)1536^+/−^ #5302 mouse infected with RML, or the large CerPrP^Sc^ deposits in the CNS of Tg(CerPrP)1536^+/−^ mice infected with CWD prions (Fig. 4). Comparison of the histoblot patterns in #5294 and #5295 mice with Tg(CerPrP)1536^+/−^ mouse #7263 that was infected with Cer/RML-4827 prions also revealed subtle differences in the regional distribution of CerPrP^Sc^ with, for example, relative sparing of the corpus callosum in the #5294 and #5295 mice infected with PMCA Cer/RML prions (Fig. 7). These differences in CerPrP^Sc^ distribution suggest that subtle strain differences distinguish Cer/RML-4827 and PMCA Cer/RML prions.

Collectively, the similar rapid incubation times of PMCA Cer/RML and Cer/RML-4827 prions, and the distinctive properties of the resulting CerPrP^Sc^, demonstrate that both processes produced novel cervid prions with biological properties distinct from CWD. Our findings therefore indicate that serial PMCA substituted for the long-term process of RML prion adaptation in Tg(CerPrP)1536^+/−^ mice. Nonetheless, our histoblotting and immunohistochemical analyses show that adaptation of RML prions in Tg(CerPrP)1536^+/−^ mice resulted in the formation of at least two distinct isolates, and that PMCA adaptation likely resulted in a third. These observations indicate that multiple isolates with different strain properties may be produced during the process of prion adaptation following inter-species transmission.

## Discussion

### Accelerated high fidelity intra-species prion amplification

The studies reported here are significant in showing that PrP^Sc^ and CWD prion infectivity from diseased deer brain are faithfully reproduced in vitro by PMCA using CerPrP^C^ from the brains of Tg(CerPrP)1536^+/−^ mice as the substrate for amplification. They extend previous reports using Tg(CerPrP)1536^+/−^ mice [44] by showing that PMCA-derived CWD prions induce disease and the production of CerPrP^Sc^ in Tg(CerPrP) mice as efficiently as prions isolated from the CNS of deer with CWD.

Tg mice represent a convenient, controlled source of PrP^C^ for PMCA with significant advantages over PrP^C^ from animals or humans. Any form of transgene-derived PrP, either mutated or PrP^C^ from different species, can be readily overexpressed on a *Prnp^0/0^* background, and the brains of Tg mice can be appropriately prepared for use in PMCA. Underscoring this concept, brain homogenate from Tg mice expressing human PrP was recently used to amplify PrP^Sc^ from the brains of variant CJD patients by PMCA [45]. As we show here, Tg mice also provide a crucial additional resource in which to fully characterize the biological properties of PMCA-derived prions. These studies raise the prospect of using PMCA and Tg mice expressing mutant and wild type PrP, and polymorphic variants thereof, from cervids, humans, cattle, sheep, rodents, horses, and other mammals, to characterize the strain and host-range properties of naturally occurring prion strains.

Our studies not only reassuringly support previous demonstrations that serial PMCA reproduces experimentally-adapted scrapie 263K prions [38],[46], but also demonstrate (to our knowledge for the first time) cell-free amplification of naturally occurring prion infectivity. Previous serial PMCA of the 263K isolate resulted in the generation of prions with apparently lower specific infectivity than brain-derived infectious material [38],[46]. Here we show that the mean incubation times of naturally occurring 04-22412 CWD and PMCA-derived CWD prion preparations in Tg(CerPrP)1536^+/−^ mice were comparable. Since each inoculum comprised similar amounts of CerPrP^Sc^, this suggests that equivalent levels of CWD prion infectivity were present in each case. While the reason for the discrepant behavior of 263K and 04-22412 CWD prions is unknown, the suggestion that PMCA may have generated a different prion strain after repeated in vitro amplification of 263K prions [38] appears unlikely in the case of PMCA of CWD prions.

To analyze and compare the strain properties of PMCA CWD and naturally occurring CWD prions we analyzed several independent criteria previously used to characterize prion strains. These included the induction of clinical signs in mice, the electrophoretic migration and glycoprofiles of CerPrP^Sc^ by Western blotting, PrP^Sc^ deposition by histoblot, cerebral vacuolization and PrP^Sc^ deposition by immunohistochemistry, and the denaturation characteristics of PrP^Sc^. Based on these criteria, it appears that PMCA CWD prions retain the biological and biochemical properties of the originating CWD prions. However, we realize that each approach is limited in its ability to unequivocally define strain variation. For example, subtle differences in the pattern of PrP^Sc^ deposition in histoblots of individual mice may result from slight variances in the locations of coronal sections between mice, or from the times at which mice were sacrificed. The sensitive and specific paraffin-embedded tissue (PET) blot technique [47] may provide finer resolution for future comparative analyses. Furthermore, while the indistinguishable denaturation profiles and GdnHCl_1/2_ values of CerPrP^Sc^ in the brains of Tg mice dying from infection with naturally occurring or PMCA-derived CWD prions suggests comparable CerPrP^Sc^ structures, equivalent conformational stability does not necessarily indicate invariant conformations at all structural levels. Other approaches may reveal evidence of PrP^Sc^ structural differences. For example, infrared-spectroscopy distinguished the secondary structures of protease-resistant PrP from two hamster scrapie strains when immunobiochemical typing failed to detect differences [48]. Fourier transform infrared-spectroscopy was also used to compare secondary structures of PMCA-generated and brain-derived protease-resistant PrP [38] as well as protease-resistant PrP products from seeded polymerization of recombinant PrP (rPrP-PMCA) [49].

### Production of novel strains following abrogation of prion transmission barriers

Strain adaptation experiments, traditionally performed in vivo, often require years to generate prions with stable biological properties. While investigating the susceptibility of Tg mice to prions from other species provides a feasible approach to address the potential for inter-species prion transmission, the studies reported here demonstrate that abrogating the barrier to mouse RML prion transmission in Tg(CerPrP) mice required several hundred days followed by strain stabilization after serial passage. Our previous studies showed that Tg(CerPrP)1536^+/−^ are also susceptible to sheep SSBP/1 scrapie prions, but with apparently less of a transmission barrier than mouse RML prions [32].

An important sequel to inter-species prion transmission is frequently the acquisition of new strain properties. The different patterns of CerPrP^Sc^ deposition in diseased Tg(CerPrP)1536^+/−^ mice following RML infection indicates that abrogation of this transmission barrier most likely results in the formation and propagation of different prion isolates in individual mice. In this case we observed two general patterns by histoblotting and immunohistochemistry: diffuse CerPrP^Sc^ deposition in mice #4825 and #5297; and small plaque deposits in the case of mice #5302 and #5300. Materials from the histoblotted #4825 and #5302 mice were not available for serial transmission studies. At the time of writing, serial transmissions of prions from the brains of mice #5297 and #5300 are ongoing. The brain of mouse #4827 was used for serial transmission and full strain characterization in Tg(CerPrP)1536^+/−^ mice. The substantial reduction and consistent time to onset of disease following serial passage of Cer/RML-4827 prions is characteristic of prion adaptation following transit across a species barrier. The ∼150 d mean incubation period of Cer/RML-4827 prions is ∼100 days shorter than either CWD or PMCA-generated CWD prions (Fig. 2), indicating adaptation of RML in Tg(CerPrP)1536^+/−^ mice and the production of novel cervid prions with biological properties distinct from CWD. Additional detailed comparisons with Tg(CerPrP) mice infected with either naturally occurring or PMCA CWD prions were consistent with the notion that the biological properties of Cer/RML-4827 prions were distinct from CWD. In accordance with previous studies of experimentally-adapted hamster prion isolates in Tg mice expressing artificial chimeric PrP genes, which indicated that a change in the conformation of PrP^Sc^ accompanied the emergence of a new prion strain [11], the conformational stability of PrP^Sc^ changed following passage of mouse RML prions from wild type mice and subsequent adaptation to form Cer/RML-4827 prions in Tg(CerPrP)1536^+/−^ mice (Fig. 6). Also in accordance with the process of prion adaptation, the profile of RML PrP^Sc^ glycosylation changed following transmission to Tg(CerPrP)1536^+/−^ mice (Fig. 3), and there were distinct differences in the morphology and neuroanatomical distribution of PrP^Sc^ in Tg(CerPrP)1536^+/−^ mice infected with Cer/RML-4827 and CWD prions.

We show that the adaptation of mouse RML prions, which required two passages in Tg(CerPrP) mice, can be accomplished by in vitro amplification of CerPrP^C^ with heterologous RML prions to create cervid adapted RML prions in a matter of weeks. Accompanying this adaptation, the RML glycopattern changed from predominantly mono- to diglycosylated PrP^Sc^, which is the form of CerPrP^Sc^ propagated in Tg(CerPrP) mice infected with RML, Cer/RML-4827 prions, and PMCA Cer/RML prions. Whereas 22 rounds were used to ensure the elimination of residual prion seed in the initial round of PMCA, it seems likely that PMCA-mediated inter-species transmissions can be accomplished with many fewer rounds of serial PMCA. Whether PMCA-mediated adaptation occurs with structural intermediates similar to the process in vivo is currently not known; however, since the properties of the initiating and resulting prions are, in most cases, likely to be distinct, it should be possible to determine the kinetics of prion adaption at each PMCA round. Should it be possible to reproducibly manipulate the extent of prion adaptation by varying the number of rounds of serial PMCA, then mechanistic studies of prion adaptation following inter-species transmission are likely to be considerably expedited by this approach.

Our studies convincingly show that PMCA of murine RML prions using Tg(CerPrP)1536^+/−^ brain homogenate generates a novel strain of cervid-adapted prions with properties distinct from either naturally occurring or PMCA-generated CWD prions. It currently is less clear whether the PrP^Sc^ structures and strain properties of amplified and in vivo derived prions are equivalent. Direct comparisons of the strain properties of PMCA Cer/RML cervid prions and in vivo-adapted strains are complicated by our observations that in vivo adaptation gives rise to individual isolates with different strain-related properties, at least as judged by histoblot and immunohistochemical profiles of PrP^Sc^. While certain strain-related attributes, including comparably rapid prion incubation times, and similar denaturation profiles of CerPrP^Sc^ after infection, suggest shared biological properties between Cer/RML-4827 and PMCA Cer/RML prions, other differences, including targeting of cerebral PrP^Sc^ deposition of Tg(CerPrP)1536^+/−^ mice infected with Cer/RML-4827 and PMCA Cer/RML prions, point to divergent strain properties and thus would rather argue for different strains. Whether inter-species PMCA-mediated prion adaptation also results in the generation of multiple and distinct prion strains remains to be determined, but our limited analyses consisting of uniform histoblot profiles, reproducible onsets of disease, and similar conformation stabilities of CerPrP^Sc^ in individual infected mice may indicate that PMCA selectively and stably propagates distinct strains following abrogation of a species barrier.

Finally, we note that under certain conditions, PMCA may result in the spontaneous formation of PK-resistant PrP species [40] and de novo generated infectivity under conditions that do not involve seeding with infectious prions [50]. While it would be of considerable interest to determine the biological properties of spontaneously-produced cervid prions by PMCA of CerPrP^C^, we feel that the possibility of spontaneous generation of infectivity in the context of the current studies is remote. The experiments of Deleault and co-workers using purified PrP^C^ plus poly(A) RNA, indicated that spontaneous generation of PrP^Sc^ was a stochastic and relatively infrequent event, estimated at <1 conversion event per 6×10^11^ input PrP^C^ molecules per PMCA round. Consequently, amplification of preexisting PrP^Sc^ molecules was considered an unlikely origin for PrP^Sc^ formation under these conditions. In contrast, in the studies reported here where PMCA reactions were seeded with either CWD or RML prions, high levels of protease-resistant PrP were amplified at each round of serial PMCA and remained consistently so during both intra- and inter-species PMCA-mediated prion amplification (Fig. 1A and B). In control experiments using unseeded healthy Tg(CerPrP)1536^+/−^ brain extract, 10 passages of serial PMCA failed to generate protease-resistant PrP (data not shown). While we have also spontaneously generated PrP^Sc^ without the addition of prion seeds (Soto and co-workers, unpublished results; Castilla and co-workers, unpublished results), the PMCA conditions required to accomplish this required modification from the standard PMCA conditions used in the current and previous studies. Standard serial PMCA conditions in which healthy hamster brain homogenate was serially diluted into itself in the absence of prion seed failed to produce protease-resistant PrP following the same number of PMCA cycles which resulted in amplification of hamster 263K prions [38]. In a larger experiment, samples of healthy brain homogenate from 10 different mice and hamsters were subjected to serial rounds of PMCA amplification in the absence of PrP^Sc^ seed using the PMCA conditions used in study. Following 20 rounds of serial PMCA, we did not observe de novo formation of PrP^Sc^, nor did these materials, when inoculated into wild-type animals, induce disease after >400 d (Soto and co-workers, unpublished observations). For these reasons we feel that the generation of PrP^Sc^ and cervid prions reported in the present study, when normal brain homogenate from Tg(CerPrP)1536^+/−^ mice was mixed with prion seeds, is unlikely to be influenced by spontaneous, de novo generated infectivity.

## Materials and Methods

### Preparation of tissue homogenates for PMCA

Healthy Tg(CerPrP)1536^+/−^ mice were perfused with phosphate-buffered saline (PBS) plus 5 mM EDTA immediately prior to harvesting the tissue. Ten % brain homogenates (w/v) were prepared in conversion buffer which consisted of PBS containing NaCl 150 mM, 1.0% Triton X-100, and the complete™ cocktail of protease inhibitors (Roche, Mannheim, Germany). The samples were clarified by a brief, low-speed centrifugation (1500 rpm for 30 s) using an Eppendorf centrifuge (Hamburg, Germany).

### Serial replication of prions in vitro by PMCA

A 1∶10 dilution of 10% brain homogenate from clinically sick 04-22412 infected mule deer or RML infected mice was diluted into a 10% brain homogenate from Tg(CerPrP)1536^+/−^ mice. Samples in 0.2 ml PCR tubes were positioned on an adaptor placed on the plate holder of a microsonicator (Misonix Model 3000, Farmingdale, NY). Each PMCA cycle consisted of 30 min incubation at 37°C followed by a 20 s pulse of sonication set at potency of 7. Samples were incubated without shaking immersed in the water of the sonicator bath. After a round of 36 cycles, a 10 µl aliquot of the amplified material was diluted into 90 µl of additional Tg(CerPrP)1536^+/−^ mouse brain homogenate and a new round of 36 PMCA cycles was performed. This procedure was repeated for 22 rounds. The detailed protocol for PMCA, including reagents, solutions and troubleshooting, has been published elsewhere [39], [51]–[53].

### Production and characterization of transgenic mice, and sources and preparation of inocula

Tg mice expressing deer PrP, referred to as Tg(CerPrP)1536 have been described previously [30]. While we showed that CWD prion incubation times are more rapid in Tg(CerPrP)1536 homozygous for the transgene array than hemizygous Tg(CerPrP)1536 mice [30], because of difficulties associated with breeding homozygous Tg(CerPrP)1536 mice we have maintained this line in the hemizygous state by breeding with *Prnp^0/0^* mice. Such mice are therefore referred to as Tg(CerPrP)1536^+/−^ mice.

CWD prions were derived from a diseased female mule deer, referred to as 04-22412 UWVS ESW/JEJ, but abbreviated here as 04-22412. The animal was homozygous for the polymorphic codon 225, encoding serine at this location. The RML isolate was originally a kind gift from Byron Caughey (Laboratory of Persistent Viral Diseases, Rocky Mountain Laboratories, Hamilton, MT) and was passaged by intracerebral inoculation of inbred FVB/N mice at the University of Kentucky.

Ten % (w/v) homogenates, in phosphate buffered saline (PBS) lacking calcium and magnesium ions, of cervid and mouse brains were prepared by repeated extrusion through an 18 gauge followed by a 21 gauge syringe needle.

### Determination of Incubation Periods

Groups of anesthetized mice were inoculated intracerebrally with 30 µl of 1% (w/v) brain extracts prepared and diluted in PBS, or 1% v/v of the final PMCA product diluted in PBS. Inoculated mice were diagnosed with prion disease following the progressive development of at least three signs including truncal ataxia, ‘plastic’ tail, loss of extensor reflex, difficulty righting, and slowed movement. The time from inoculation to the onset of definitive and subsequently progressive clinical signs is referred to as the incubation time.

### Analysis of PrP in CNS

For PrP analysis in brain extracts, total protein content from 10% brain homogenates prepared in PBS was determined by bicinchoninic acid (BCA) assay (Pierce Biotechnology Inc., Rockford, IL). Brain extracts were either untreated or treated with 40 µg/ml PK for one hour at 37°C in the presence of 2% sarkosyl. Protease digestion was terminated with 4 mM phenyl methyl sulfonyl fluoride (PMSF). Proteins were separated by sodium dodecyl sulfate polyacrylamide gel electrophoresis (SDS-PAGE). Proteins thus resolved were electrophoretically transferred to PVDF-FL membranes (Millipore, Billerica, MA). Membranes were probed with mAb 6H4 [54], or the Hum-P anti-PrP recombinant Fab [55] followed by horse radish peroxidase-conjugated sheep anti-mouse IgG or goat anti-human secondary antibody respectively. Signal was developed using ECL-plus detection (Amersham), and analyzed using a FLA-5000 scanner (Fuji).

Histoblots of 10 µm thick cryostat sections were generated and transferred to nitrocellulose as previously described [41]. Histoblots were immunostained with the Hum-P anti-PrP recombinant Fab followed by alkaline phosphatase-conjugated goat anti-human secondary antibody. Images were captured using a Nikon SM21000 microscope with Photometrics Coolsnap CF digital imager and processed using MetaMorph software

The unfolding characteristics of PrP^Sc^ in brain homogenates of terminally sick mice were analyzed using a Western blot-based conformational stability assay [12],[32],[43] which is a modification of the original ELISA based protocol [11].

Analysis of PrP in the brains of Tg mice by immunohistochemistry was performed as previously described [56] using anti-PrP mAb 6H4 [54] as primary antibody and IgG_1_ biotinylated goat anti-mouse secondary antibody (Southern Biotech). Digitized images for figures were obtained by light microscopy using a Nikon Eclipse E600 microscope equipped with a Nikon DMX 1200F digital camera.

## References

[1] Pattison IH, Gajdusek DC, Gibbs CJ, Alpers MP (1965). Experiments with scrapie with special reference to the nature of the agent and the pathology of the disease.. Slow, Latent and Temperate Virus Infections, NINDB Monograph 2.

[2] Scott M, Foster D, Mirenda C, Serban D, Coufal F (1989). Transgenic mice expressing hamster prion protein produce species-specific scrapie infectivity and amyloid plaques.. Cell.

[3] Prusiner SB, Scott M, Foster D, Pan K-M, Groth D (1990). Transgenetic studies implicate interactions between homologous PrP isoforms in scrapie prion replication.. Cell.

[4] Scott M, Groth D, Foster D, Torchia M, Yang S-L (1993). Propagation of prions with artificial properties in transgenic mice expressing chimeric PrP genes.. Cell.

[5] Telling GC, Scott M, Hsiao KK, Foster D, Yang SL (1994). Transmission of Creutzfeldt-Jakob disease from humans to transgenic mice expressing chimeric human-mouse prion protein.. Proc Natl Acad Sci U S A.

[6] Telling GC, Scott M, Mastrianni J, Gabizon R, Torchia M (1995). Prion propagation in mice expressing human and chimeric PrP transgenes implicates the interaction of cellular PrP with another protein.. Cell.

[7] Kocisko DA, Come JH, Priola SA, Chesebro B, Raymond GJ (1994). Cell-free formation of protease-resistant prion protein.. Nature.

[8] Bessen RA, Marsh RF (1994). Distinct PrP properties suggest the molecular basis of strain variation in transmissible mink encephalopathy.. J Virol.

[9] Telling GC, Parchi P, DeArmond SJ, Cortelli P, Montagna P (1996). Evidence for the conformation of the pathologic isoform of the prion protein enciphering and propagating prion diversity.. Science.

[10] Korth C, Kaneko K, Groth D, Heye N, Telling G (2003). Abbreviated incubation times for human prions in mice expressing a chimeric mouse-human prion protein transgene.. Proc Natl Acad Sci U S A.

[11] Peretz D, Williamson RA, Legname G, Matsunaga Y, Vergara J (2002). A change in the conformation of prions accompanies the emergence of a new prion strain.. Neuron.

[12] Scott MR, Peretz D, Nguyen HO, Dearmond SJ, Prusiner SB (2005). Transmission barriers for bovine, ovine, and human prions in transgenic mice.. J Virol.

[13] Hill AF, Joiner S, Wadsworth JD, Sidle KC, Bell JE (2003). Molecular classification of sporadic Creutzfeldt-Jakob disease.. Brain.

[14] Gambetti P, Kong Q, Zou W, Parchi P, Chen SG (2003). Sporadic and familial CJD: classification and characterisation.. Br Med Bull.

[15] Bartz JC, Marsh RF, McKenzie DI, Aiken JM (1998). The host range of chronic wasting disease is altered on passage in ferrets.. Virology.

[16] Bartz JC, Bessen RA, McKenzie D, Marsh RF, Aiken JM (2000). Adaptation and selection of prion protein strain conformations following interspecies transmission of transmissible mink encephalopathy.. J Virol.

[17] Wilesmith JW, Wells GAH, Cranwell MP, Ryan JBM (1988). Bovine spongiform encephalopathy: epidemiological studies.. Vet Rec.

[18] Wyatt JM, Pearson GR, Smerdon TN, Gruffydd-Jones TJ, Wells GAH (1991). Naturally occurring scrapie-like spongiform encephalopathy in five domestic cats.. Vet Rec.

[19] Kirkwood JK, Cunningham AA (1994). Epidemiological observations on spongiform encephalopathies in captive wild animals in the British Isles.. Vet Rec.

[20] Collinge J, Sidle KCL, Meads J, Ironside J, Hill AF (1996). Molecular analysis of prion strain variation and the aetiology of “new variant” CJD.. Nature.

[21] Hill AF, Desbruslais M, Joiner S, Sidle KCL, Gowland I (1997). The same prion strain causes vCJD and BSE.. Nature.

[22] Bruce ME, Will RG, Ironside JW, McConnell I, Drummond D (1997). Transmissions to mice indicate that ‘new variant’ CJD is caused by the BSE agent.. Nature.

[23] Scott MR, Safar J, Telling G, Nguyen O, Groth D (1997). Identification of a prion protein epitope modulating transmission of bovine spongiform encephalopathy prions to transgenic mice.. Proc Natl Acad Sci U S A.

[24] Marsh RF, Bessen RA, Lehmann S, Hartsough GR (1991). Epidemiological and experimental studies on a new incident of transmissible mink encephalopathy.. J Gen Virol.

[25] Benestad SL, Sarradin P, Thu B, Schonheit J, Tranulis MA (2003). Cases of scrapie with unusual features in Norway and designation of a new type, Nor98.. Vet Rec.

[26] Le Dur A, Beringue V, Andreoletti O, Reine F, Lai TL (2005). A newly identified type of scrapie agent can naturally infect sheep with resistant PrP genotypes.. Proc Natl Acad Sci U S A.

[27] Simmons MM, Konold T, Simmons HA, Spencer YI, Lockey R (2007). Experimental transmission of atypical scrapie to sheep.. BMC Vet Res.

[28] Saborio GP, Permanne B, Soto C (2001). Sensitive detection of pathological prion protein by cyclic amplification of protein misfolding.. Nature.

[29] Telling G (2001). Protein-based PCR for prion diseases?. Nat Med.

[30] Browning SR, Mason GL, Seward T, Green M, Eliason GA (2004). Transmission of prions from mule deer and elk with chronic wasting disease to transgenic mice expressing cervid PrP.. J Virol.

[31] Angers RC, Browning SR, Seward TS, Sigurdson CJ, Miller MW (2006). Prions in skeletal muscles of deer with chronic wasting disease.. Science.

[32] Green KM, Browning SR, Seward TS, Jewell JE, Ross DL (2008). The elk PRNP codon 132 polymorphism controls cervid and scrapie prion propagation.. Journal of General Virology.

[33] Meade-White K, Race B, Trifilo M, Bossers A, Favara C (2007). Resistance to chronic wasting disease in transgenic mice expressing a naturally occurring allelic variant of deer prion protein.. J Virol.

[34] LaFauci G, Carp RI, Meeker HC, Ye X, Kim JI (2006). Passage of chronic wasting disease prion into transgenic mice expressing Rocky Mountain elk (Cervus elaphus nelsoni) PrPC.. J Gen Virol.

[35] Tamguney G, Giles K, Bouzamondo-Bernstein E, Bosque PJ, Miller MW (2006). Transmission of elk and deer prions to transgenic mice.. J Virol.

[36] Kong Q, Huang S, Zou W, Vanegas D, Wang M (2005). Chronic wasting disease of elk: transmissibility to humans examined by transgenic mouse models.. J Neurosci.

[37] Trifilo MJ, Ying G, Teng C, Oldstone MB (2007). Chronic wasting disease of deer and elk in transgenic mice: Oral transmission and pathobiology.. Virology.

[38] Castilla J, Saa P, Hetz C, Soto C (2005). In vitro generation of infectious scrapie prions.. Cell.

[39] Bieschke J, Weber P, Sarafoff N, Beekes M, Giese A (2004). Autocatalytic self-propagation of misfolded prion protein.. Proc Natl Acad Sci U S A.

[40] Murayama Y, Yoshioka M, Yokoyama T, Iwamaru Y, Imamura M (2007). Efficient in vitro amplification of a mouse-adapted scrapie prion protein.. Neurosci Lett.

[41] Taraboulos A, Jendroska K, Serban D, Yang S-L, DeArmond SJ (1992). Regional mapping of prion proteins in brains.. Proc Natl Acad Sci USA.

[42] Hecker R, Taraboulos A, Scott M, Pan K-M, Torchia M (1992). Replication of distinct prion isolates is region specific in brains of transgenic mice and hamsters.. Genes Dev.

[43] Xie Z, O'Rourke KI, Dong Z, Jenny AL, Langenberg JA (2006). Chronic wasting disease of elk and deer and Creutzfeldt-Jakob disease: comparative analysis of the scrapie prion protein.. J Biol Chem.

[44] Kurt TD, Perrott MR, Wilusz CJ, Wilusz J, Supattapone S (2007). Efficient in vitro amplification of chronic wasting disease PrPRES.. J Virol.

[45] Jones M, Peden AH, Prowse CV, Groner A, Manson JC (2007). In vitro amplification and detection of variant Creutzfeldt-Jakob disease PrPSc.. J Pathol.

[46] Weber P, Giese A, Piening N, Mitteregger G, Thomzig A (2006). Cell-free formation of misfolded prion protein with authentic prion infectivity.. Proc Natl Acad Sci U S A.

[47] Schulz-Schaeffer WJ, Tschoke S, Kranefuss N, Drose W, Hause-Reitner D (2000). The paraffin-embedded tissue blot detects PrP(Sc) early in the incubation time in prion diseases.. Am J Pathol.

[48] Thomzig A, Spassov S, Friedrich M, Naumann D, Beekes M (2004). Discriminating scrapie and bovine spongiform encephalopathy isolates by infrared spectroscopy of pathological prion protein.. J Biol Chem.

[49] Atarashi R, Moore RA, Sim VL, Hughson AG, Dorward DW (2007). Ultrasensitive detection of scrapie prion protein using seeded conversion of recombinant prion protein.. Nat Methods.

[50] Deleault NR, Harris BT, Rees JR, Supattapone S (2007). Formation of native prions from minimal components in vitro.. Proc Natl Acad Sci U S A.

[51] Castilla J, Saá P, Soto C, Lehmann S, Grassi J (2004). Cyclic Amplification of Prion Protein Misfolding.. Methods and Tools in Bioscience and Medicine. Techniques in Prion Research..

[52] Castilla J, Saa P, Morales R, Abid K, Maundrell K (2006). Protein misfolding cyclic amplification for diagnosis and prion propagation studies.. Methods Enzymol.

[53] Saa P, Castilla J, Soto C, Sigurdsson EM (2004). Cyclic Amplification of Protein Misfolding and Aggregation.. Amyloid Proteins: Methods and Protocols..

[54] Korth C, Stierli B, Streit P, Moser M, Schaller O (1997). Prion (PrP^Sc^)-specific epitope defined by a monoclonal antibody.. Nature.

[55] Safar JG, Scott M, Monaghan J, Deering C, Didorenko S (2002). Measuring prions causing bovine spongiform encephalopathy or chronic wasting disease by immunoassays and transgenic mice.. Nat Biotechnol.

[56] Muramoto T, DeArmond SJ, Scott M, Telling GC, Cohen FE (1997). Heritable disorder resembling neuronal storage disease in mice expressing prion protein with deletion of an alpha-helix.. Nat Med.

